# A retrospective study on the management of massive hemoptysis by bronchial artery embolization: risk factors associated with recurrence of hemoptysis

**DOI:** 10.1186/s12890-023-02371-1

**Published:** 2023-03-16

**Authors:** Hui Li, Xu Ding, Shuo Zhai, Kun Gao

**Affiliations:** 1 Department of Intervention, Beijing Chao-Yang Hospital, Capital Medical University, Beijing, 100020 China; 2 Department of Vascular Surgery, Chui Yang Liu Hospital affiliated to Tsinghua University, Beijing, China; 3 Department of Radiology and Nuclear Medicine, Xuanwu Hospital, Capital Medical University, Beijing, China

**Keywords:** Massive hemoptysis, Bronchial artery embolization, Retrospective study

## Abstract

**Background:**

Massive hemoptysis is a life-threatening condition that requires immediate treatment. This study aimed to retrospectively analyze the outcome of bronchial artery embolization (BAE) for massive hemoptysis, as well as potential factors that may contribute to the recurrence of hemoptysis after BAE.

**Methods:**

A total of 105 patients with massive hemoptysis treated with BAE were analyzed.

**Results:**

The immediate control rate of bleeding was 84.8% (67/79); however, during the 36-month follow-up, 45.3% (29 out of 64) of the patients had recurrent hemoptysis after BAE. Comorbidities, pituitary hormone treatment, the angiographic appearance of artery dilation and hypertrophy, and the materials used for BAE were significantly correlated with the success rate of the BAE, while lack of pituitary hormone treatment and existence of arterio-arterial or arteriovenous fistula were risk factors for the recurrence of hemoptysis after BAE. Only a small proportion of patients (9/105, 8.6%) had mild complications after BAE treatment.

**Conclusion:**

Findings suggest that BAE continues to be an effective treatment for massive hemoptysis in emergency settings. Moreover, the treatment of underlying pulmonary diseases and comorbidities is important to increase BAE’s success rate of BAE and decrease the risk of recurrent hemoptysis after BAE.

## Introduction

Massive hemoptysis is defined as the expectoration of blood from the lower respiratory tract totaling 300–600 mL over 24 h period [1, 2]. Massive hemoptysis is a life-threatening condition with mortality ranging from 6.5–38% [3–6]. The etiology of massive hemoptysis varies by region, and the most common causes are bronchiectasis, tuberculosis, lung cancer, necrotizing pneumonia, and cryptogenic hemoptysis [3, 4, 6]. The anatomic source of massive hemoptysis is predominantly the bronchial arteries (90%), and the rest is from either the pulmonary arteries (5%), aorta, or other systemic arteries [3, 7, 8].

Bronchial artery embolization (BAE) was introduced in the clinic in 1973 and now considered the first-line therapy to control bleeding from massive hemoptysis immediately [6, 9]. Currently, technical success and immediate control of bleeding with BAE are high; however, the recurrence rate of hemoptysis after BAE may vary between 10 and 55% [10–14]. A systemic review reported that recurrence of hemoptysis after BAE may be attributable to incomplete embolization, recanalization of previously embolized arteries, or recruitment of new collaterals due to underlying disease progression [6]. In this retrospective analysis, we sought to analyze the factors that may be associated with the success of BAE, and the risk factors that may contribute to the recurrence of hemoptysis after BAE.

## Materials and methods

### Case selection

This was a retrospective study on patients who met the diagnostic criteria of massive hemoptysis, 300-600 mL hemoptysis within 24 h [10, 11, 15]. Patients hospitalized due to massive hemoptysis and treated with BAE in Beijing Chaoyang Hospital from January 1^st^, 2012, to March 31^st^, 2016, were enrolled in this study. Before BAE treatment, patients were examined using high-resolution CT (HRCT), chest-enhanced CT or CT angiography (CTA), and bronchoscopy. Conservative treatment, including oxygenation and medication to control bleeding, phlegm, or infection, was administered to the patients before the BAE. Patients with hemoptysis who were not treated with BAE were excluded from the study.

### Methods

All patients underwent laboratory tests, including routine blood and coagulation function tests. To confirm the location of the bleeding, angiography of the aortic arch and thoracic aorta was performed through the right femoral artery. Depending on the location of the primary lung lesion shown by HRCT or bronchoscopy, selective angiography of the bronchial, subclavian, intercostal, and phrenic arteries was performed with a left gastric or Cobra catheter. Once the bleeding site was confirmed and following the guidelines of the Chinese Medical Association [16–18], arterial embolization was performed. Embolizing materials including micro-coils, gelatin sponge, thread, or polyvinyl alcohol (PVA 300-500 µm or 500-700 µm) were delivered via a microcatheter, which was carefully extended out to the bleeding site to avoid spinal artery injury. The success of the BAE was confirmed by post-embolization angiography showing blockade of the artery and disappearance of residual flow to the lesion.

The patients were followed up from day 2 to 60 months (5 years) after BAE. The final follow-up date was either: the date of death, the date of lobectomy by surgery, or February the 28^th^, 2017. During the follow-up, the following information was collected: date and amount of recurrent hemoptysis and the treatment; date of surgical intervention (lobectomy) and post-operative treatment; and for deceased patients, the date, time, and cause of death, and whether or not they had recurrent hemoptysis before death.

### Evaluation on therapeutic outcome

1. **Immediate effectiveness**.

Immediate effectiveness was determined by technical success and immediate control of bleeding. Technical success: The success of BAE was confirmed using post-embolization angiography. Immediate control of bleeding: The patient did not have hemoptysis within 24 h after the BAE procedure. If the patient had recurrent bleeding in the respiratory tract or fresh blood in the sputum within 24 h after the BAE, it was counted as a failed case of BAE. However, it was considered successful if the patient had brown sputum or a small amount of blood in the sputum.

2. **Long-term outcome**.

Long-term outcome of the BAE was determined by the Cumulative hemoptysis non-recurrence rates at 1, 3, 12, 24, and 36 months after BAE treatment [11]. Hemoptysis recurrence was defined as fresh hemoptysis with blood clots or fresh blood in the sputum after the disappearance of hemoptysis following BAE treatment. Early recurrence was defined as recurrence occurred within one month after BAE, and late recurrence if it occurring one month after the BAE procedure.

### Statistical analysis

Discrete variables were expressed as frequency (%), and the chi-square or Fisher’s exact probability test was used. The odds ratio (OR) of the risk factors that potentially contributed to the treatment success was analyzed by logistic regression with multifactor correction, an *a* value of 0.05 was significant. Hazard ratios (HR) of the factors possibly attributed to hemoptysis recurrence were analyzed using univariate analysis, multivariate logistic analysis, and COX multivariate analysis. All data analyses were performed using SAS 9.3 statistical analysis software. *P* < 0.05 was considered significant.

## Results

### Demographic characteristics of the patients

As shown in Table 1, 105 patients were enrolled in the study. Of these, 67 were male (64%), 38 were female (36%), and 60 (57%) under 60 years of age. Nearly half of the enrolled patients (45%) were smokers. Underlying diseases of massive hemoptysis in this study were bronchiectasis, 56 (53.3%); lung infection, 44 (41.9%); and lung tuberculosis, 15 (14.3%).

**Table 1:** Demographic information of the patients

	Level	Number (%)
Age	< 60 yr	60 (57.14)
	≥ 60 yr	45 (42.86)
Gender	Male	67 (63.81)
	Female	38 (36.19)
History of lung tuberculosis	Yes	15 (14.29)
Comorbidities^a^	Yes	14 (13.33)
Smoking	Yes	47 (44.76)
Lung infection	Yes	44 (41.90)
Bronchiectasis	Yes	56 (53.33)

^a^Comorbidities other than hypertension, diabetes or tuberculosis

### Treatment and outcomes of massive hemoptysis by the BAE

**Fig. 1:**
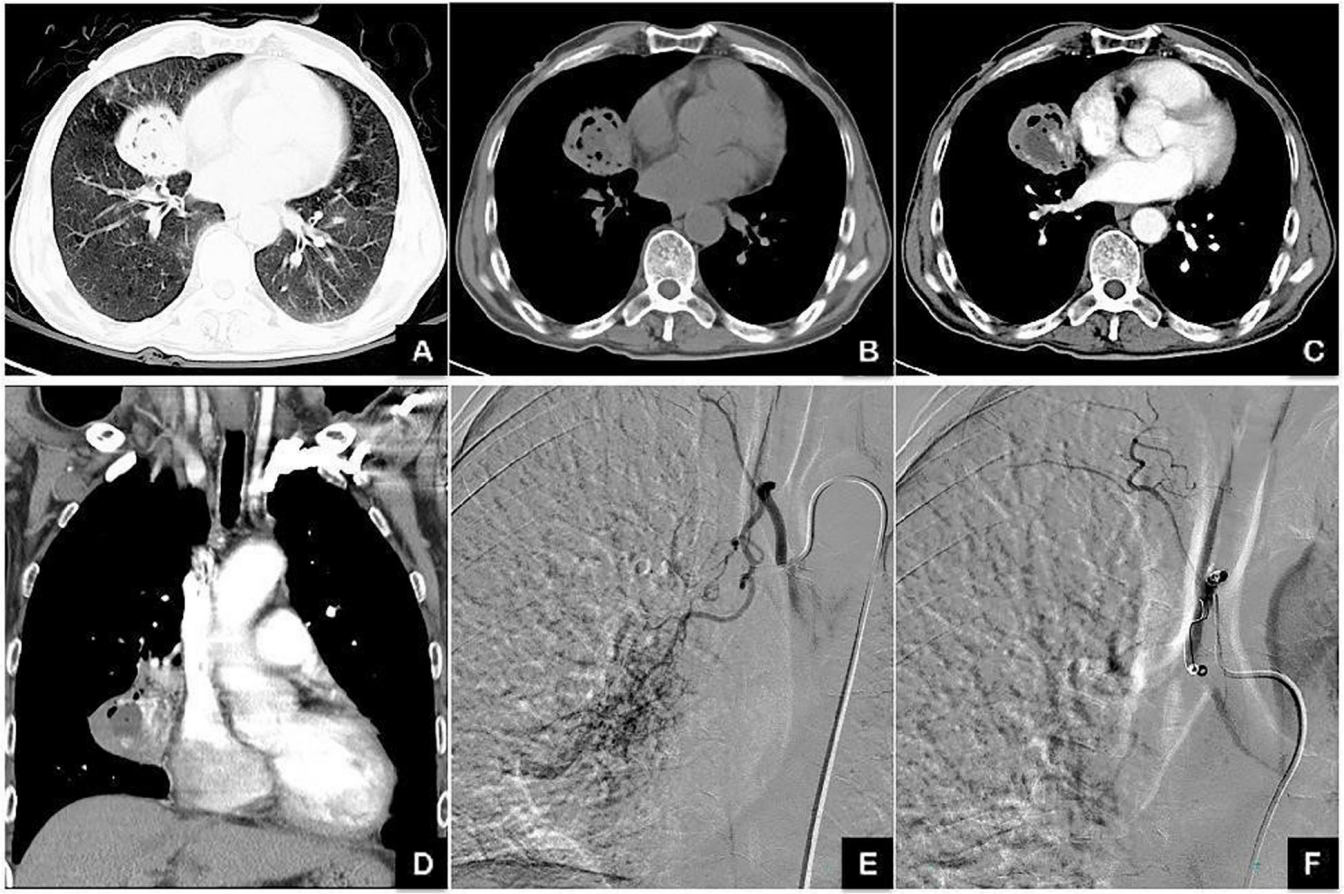
High resolution chest CT (HRCT) and digital subtraction angiogram before and after the BAE in case #1. A 62-year-old male patient had history of intermittent hemoptysis for 30 years, became worse for one month with approximately 150 mL of blood each time. **A-D**: High resolution chest CT (HRCT) images showing reduced volume and uneven density in the right middle lobe as well as cavities and necrosis. **E**: Digital subtraction angiogram after selective catheterization of the right bronchial artery and super-selective catheterization with a microcatheter, showing hypertrophied artery and hyper-vascularity in the right middle lobe; **F**: Digital subtraction angiogram after embolization, demonstrating no residual flow to the lesion

**Fig. 2:**
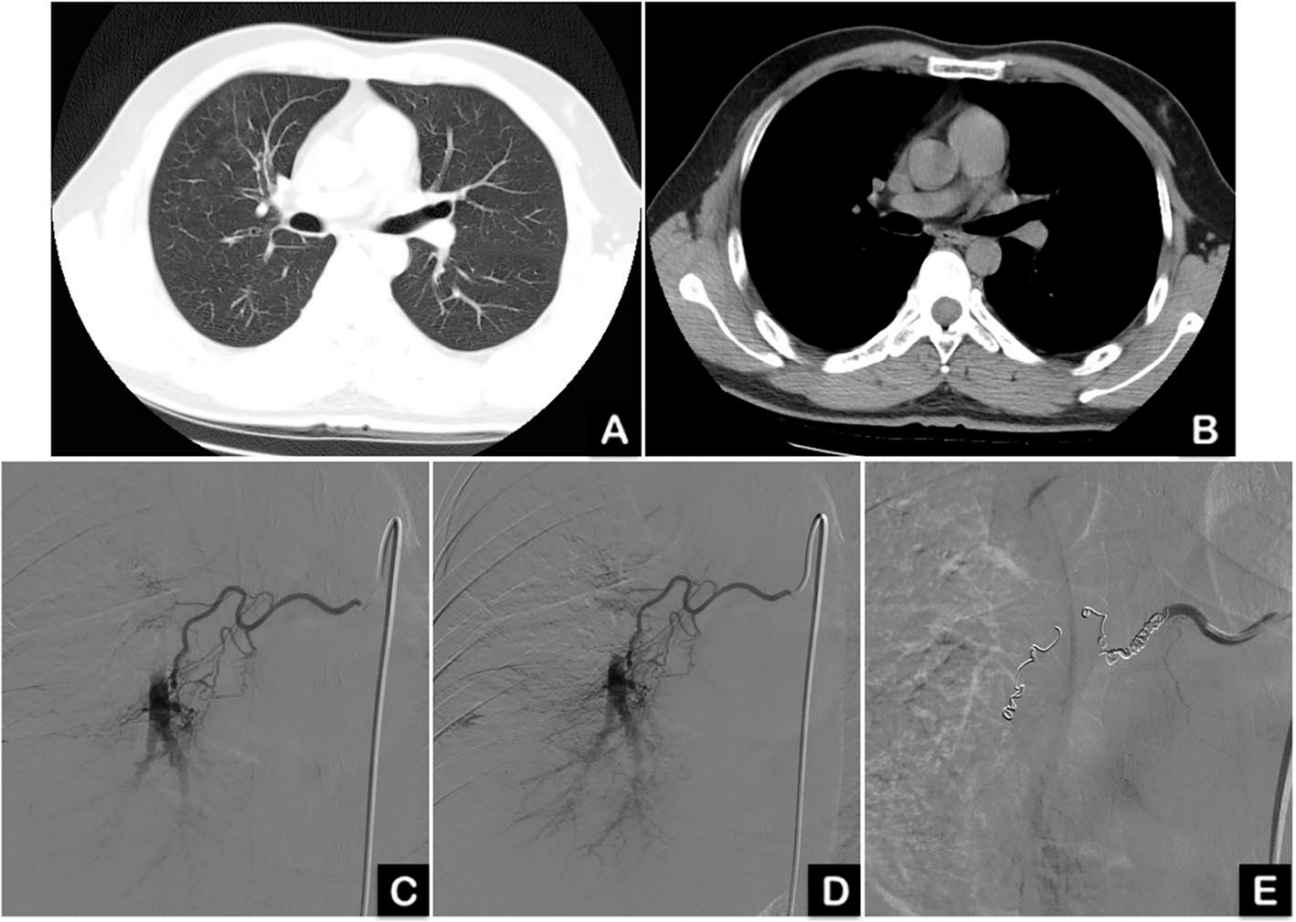
High resolution chest CT (HRCT) and digital subtraction angiogram before and after the BAE in case #2. A 33-year-old male patient with 10 years of smoking history had cryptogenic hemoptysis for one week with approximately 100 mL of blood each time. **A** and **B**: High resolution chest CT showed patchy and ground glass image in the right lung, indicating accumulation of blood in the alveoli. **C** and **D**: Digital subtraction angiograms from a selectively catheterized right bronchial artery, showing hypertrophied right bronchial artery and branches as well as hyper-vascular lesions. **E**: Digital subtraction angiogram after embolization, showing no residual flow to the lesion area

As shown in the representative case report (Figs. 1 and 2), high-resolution chest CT demonstrated lesions in the lung, and the origin of the bleeding from the bronchial artery was confirmed by angiography via selective catheterization. Successful control of bleeding was confirmed by a post-embolization angiogram showing no residual flow to the lesion area. This study’s rate of immediate bleeding control was 84.8% (67/79). Long-term follow-up of the 64 patients showed that 35 (54.7%) had hemoptysis recurrence up to 36 months after BAE. Moreover, 29 patients (45.3%) had recurrent hemoptysis after BAE treatment, with a median length of 10 months (range: 2 days to 36 months), and 93.1% of the recurrence occurred within 2 years of BAE.

Over half of the patients (58 out of 105, 55%) were embolized with coil only, and one-third (34 of 105, 32%) were treated with microsphere plus coil (Table 2). The remaining patients were embolized with other materials, including MWCE coil, PVA, gelatin sponge (Gelfoam), or thread. Ten of the 105 (9.5%) patients underwent lobectomy after BAE treatment for massive hemoptysis failed. While the majority of the patients (96 of 105, 91.4%) had no complications following BAE treatment, 9 (8.6%) patients had mild complications after the treatment, including chest pain (8 patients), nausea and gastrointestinal discomfort (1), fever (1), and aortic intramural hematoma during the BAE procedure.

**Table 2:** Treatment and outcome of massive hemoptysis by the BAE

Treatment and outcomes		Number (%)
Materials	MWCE + coil	1 ( 0.95)
	PVA + MWCE + Coil	1 ( 0.95)
	Coil + gelatin sponge	3 ( 2.86)
	Gelatin sponge + microsphere	1 ( 0.95)
	Coil embolization	58 (55.24)
	Microsphere embolization	4 ( 3.81)
	Microsphere + coil	34 (32.38)
	Coil + gelatin sponge	1 ( 0.95)
	Thread embolization	1 ( 0.95)
	Thread + PVA + coil	1 ( 0.95)
Surgical intervention after the BAE	Lobectomy	10 (9.52)
Complications	No	96 (91.43)
	Yes^a^	9 ( 8.57)

^a^8 Patients had chest pain after the procedure, one patient had aortic intramural hematoma during the procedure, one patient had fever, and 2 patients had nausea and gastrointestinal discomfort. BAE: bronchial artery embolism; PVA: polyvinyl alcohol

### Factors affecting the outcome of the BAE

First, the factors that might be associated with the success rate of BAE were analyzed. As shown in Table 3, patients with comorbidities had a significantly lower BAE success rate than patients without comorbidities, 64.3% vs 87.9% (*P* = 0.037), respectively. Similarly, patients treated with pituitary hormones had a significantly lower BAE success rate than those treated without hormone treatment, 76.4% vs 94.0% (*P* = 0.012), respectively. However, interestingly, patients with an angiographic appearance of arterial dilation and hypertrophy had a significantly higher BAE success rate than patients without, 90.2% vs 46.2%, (*P* = 0.001), respectively. In addition, patients treated with the coil in combination with other materials had a significantly higher BAE success rate than patients treated with only the coil, 93.6% vs 77.6% (*P* = 0.023) respectively. Other factors, including age, sex, smoking, underlying diseases (lung tuberculosis, infection, and bronchiectasis), angiographic tortuosity, angiographic disorder, increased angiographic staining, location of bronchial artery disease, vessel fistula, number of abnormal bronchial arteries, and bleeding amount, did not affect the BAE success rate (Table 3).

**Table 3:** Factors associated with BAE success rate

Factors	Level	Successful	Failed	Row Sub-total	*P*
Age	≥ 60 yr	38 (84.44)	7 (15.56)	45 (42.86)	0.938^a^
	< 60 yr	51 (85.00)	9 (15.00)	60 (57.14)	
Gender	Male	56 (83.58)	11 (16.42)	67 (63.81)	0.655^a^
	Female	33 (86.84)	5 (13.16)	38 (36.19)	
Hypertension	Yes	29 (87.88)	4 (12.12)	33 (31.43)	0.547^a^
	No	60 (83.33)	12 (16.67)	72 (68.57)	
Diabetes	Yes	4 (100.0)	0 ( 0.00)	4 ( 3.81)	1.000^b^
	No	85 (84.16)	16 (15.84)	101 (96.19)	
History of lung tuberculosis	Yes	14 (93.33)	1 ( 6.67)	15 (14.29)	0.458^b^
	No	75 (83.33)	15 (16.67)	90 (85.71)	
Comorbidities	Yes	9 (64.29)	5 (35.71)	14 (13.33)	**0.037**^b^
	No	80 (87.91)	11 (12.09)	91 (86.67)	
Smoking	Yes	40 (85.11)	7 (14.89)	47 (44.76)	0.930^a^
	No	49 (84.48)	9 (15.52)	58 (55.24)	
Lung infection	Yes	39 (88.64)	5 (11.36)	44 (41.90)	0.348^a^
	No	50 (81.97)	11 (18.03)	61 (58.10)	
Bronchiectasis	Yes	51 (91.07)	5 ( 8.93)	56 (53.33)	0.054^a^
	No	38 (77.55)	11 (22.45)	49 (46.67)	
Treated with pituitary hormone	Yes	42 (76.36)	13 (23.64)	55 (52.38)	**0.012**^a^
	No	47 (94.00)	3 ( 6.00)	50 (47.62)	
Angiographic dilation & hypertrophy	Yes	83 (90.22)	9 ( 9.78)	92 (87.62)	**0.001**^b^
	No	6 (46.15)	7 (53.85)	13 (12.38)	
Angiographic tortuosity	Yes	55 (82.09)	12 (17.91)	67 (63.81)	0.312^a^
	No	34 (89.47)	4 (10.53)	38 (36.19)	
Angiographic disorder	Yes	48 (88.89)	6 (11.11)	54 (51.43)	0.226^a^
	No	41 (80.39)	10 (19.61)	51 (48.57)	
Increased angiographic staining	Yes	32 (91.43)	3 ( 8.57)	35 (33.33)	0.179^a^
	No	57 (81.43)	13 (18.57)	70 (66.67)	
Bronchial artery disease	Bilateral Right	32 (76.19) 44 (89.80)	10 (23.81) 5 (10.20)	42 (40.00) 49 (46.67)	0.131^a^
	Left	13 (92.86)	1 ( 7.14)	14 (13.33)	
Arterio-arterial or arteriovenous fistula	No	52 (81.25)	12 (18.75)	64 (60.95)	0.211^a^
	Yes	37 (90.24)	4 ( 9.76)	41 (39.05)	
Number abnormal bronchial artery	One	35 (85.37)	6 (14.63)	41 (39.05)	0.890^a^
	≥ 2	54 (84.38)	10 (15.63)	64 (60.95)	
Hemoptysis amount	≥ 300 mL < 300 mL	55 (83.33) 34 (87.18)	11 (16.67) 5 (12.82)	66 (62.86) 39 (37.14)	0.596^a^
Materials for embolization	Other materials	44 (93.62)	3 ( 6.38)	47 (44.76)	**0.023**^a^
	Coil	45 (77.59)	13 (22.41)	58 (55.24)	

^a^Chi-square

^b^Fisher’s Exact Test

Next, risk factors that may be attributed to the failure of the procedure within 24 h after BAE were analyzed. As shown in Table 4, before correction of the logistic regression, the OR, 95% CI) was as follows: comorbidities:4.041 (1.144–14.271), *P* = 0.030; pituitary hormone treatment:4.849 (1.292–18.199), *P* = 0.019; the angiographic appearance of artery dilation and hypertrophy:0.093 (0.026–0.337), *P* = 0.000; materials for the BAE:0.236 (0.063–0.886), *P* = 0.032. After the correction, the OR (95% CI) was7.269 (1.597–33.074), *P* = 0.010; pituitary hormone treatment:4.452 (1.059–18.717), *P* = 0.042; angiographic dilation and hypertrophy:0.068 (0.015–0.300), *P* = 0.000 (Table 4). These findings suggest that comorbidities, treatment with pituitary hormone, the angiographic appearance of artery dilation and hypertrophy, and materials used for the BAE, were factors attributing to the failure of BAE within 24 h.

**Table 4:** Risk factors associated with failure of the BAE procedure

Independent variables	Level of variables	Uncorrected		After correction	
		OR^a^ (95%CI)	*P*	OR^a^(95%CI)	*P*
Comorbidities	Yes vs No	4.041(1.144–14.271)	0.030	7.269(1.597–33.074)	0.010
Treated with pituitary hormone	Yes vs No	4.849(1.292–18.199)	0.019	4.452(1.059–18.717)	0.042
Angiographic dilation & thickening	Yes vs No	0.093(0.026–0.337)	0.000	0.068(0.015–0.300)	0.000
Materials^b^	Other materials vs coil only	0.236(0.063–0.886)	0.032	-	-

^a^Since the univariate analysis of “comorbidities, treated with pituitary hormone, angiographic dilation & thickening, and materials” indicated significant difference, multivariate logistic analysis was performed, and independent variable was screened by stepwise regression

^b^This variable was excluded from multifactor correction. Comorbidities, treated with pituitary hormone, and angiographic dilation & thickening were adjusted

The risk factors that may contribute to the recurrence of hemoptysis after BAE were also analyzed. As shown in Table 5, the uncorrected and corrected HR, 95% CI) in the patients without pituitary hormone treatment were 2.278 (1.058–4.904), *P* = 0.035, and 2.057 (0.958–4.462), *P* = 0.068, respectively. In contrast, uncorrected and corrected HR (95% CI) in the patients with arterio-arterial or arteriovenous fistula were 2.321 (1.108–4.863), *P* = 0.026, and 2.103 (0.997–4.436), *P* = 0.051, respectively (Table 5). This suggests that pituitary hormone treatment and vessel fistula may be associated with the long-term outcome of BAE treatment in patients with massive hemoptysis.

**Table 5:** Risk factors associated with recurrence of hemoptysis after BAE

Independent variables	Level of variables	Uncorrected		After correction	
		HR (95%CI)	*P*	HR ^a^(95%CI)	*P*
Treated with pituitary hormone	No vs Yes	2.278(1.058–4.904)	0.035	2.057(0.948–4.462)	0.068
Arterio-arterial or arteriovenous fistula	Yes vs No	2.321(1.108–4.863)	0.026	2.103(0.997–4.436)	0.051

^a^Since the univariate analysis of “treated with pituitary hormone” and “arterio-arterial or arteriovenous fistula” indicated significant difference, they were further corrected by COX multivariate analysis. “Treated with pituitary hormone” and “arterio-arterial or arteriovenous fistula” were adjusted

## Discussion

BAE is an established procedure for the treatment of massive and life-threatening hemoptysis. In this retrospective study, the etiology of massive hemoptysis was bronchiectasis in over half (53.3%) of the enrolled patients and more than half (55%) of the patients were only treated with coils during the BAE procedure. The clinical effectiveness of the BAE was 90.5% (95/105), but BAE failed in 10 out of 105 patients (9.5%) who were treated with surgical intervention (lobectomy). Comorbidities, pituitary hormone treatment, the angiographic appearance of arterial dilation and thickening, and materials used for BAE might affect the success rate of BAE. In contrast, arterio-artery or arteriovenous fistula and pituitary hormone treatment were risk factors for the recurrence of hemoptysis after BAE treatment. Most patients (91%) had no complications after the BAE procedure in this study.

Massive hemoptysis is defined as the expectoration of > 200 mL over 24-h. The mortality rate of massive and untreated hemoptysis is greater than 50% [19]. Massive hemoptysis is caused by asphyxiation, rather than exsanguination. In addition, respiratory distress due to massive hemoptysis is not only dependent on the volume of bleeding but also on underlying comorbidities [6]. Lung tuberculosis is the most common cause of massive hemoptysis, followed by other lung diseases including lung cancer, bronchiectasis, cystic fibrosis, alveolar hemorrhage syndromes, lung abscess, and trauma [3, 8, 10, 20, 21]. In the current study, the most common etiology of massive hemoptysis was bronchiectasis (53.3%), followed by lung infection (41.9%) and pulmonary tuberculosis (14.3%). Consistent with our findings, a report from the Mayo Clinic of 54 patients treated with BAE observed that the etiologies for hemoptysis were bronchiectasis, pulmonary hypertension, malignancy, mycetoma, and other causes [22].

BAE is a minimally invasive procedure that embolizes the bronchial arterial system. It is the main and most important therapeutic procedure for controlling massive hemoptysis. However, BAE is not uniformly successful, and therapeutic results from BAE vary in previous reports [5, 6, 11, 23–26]. In this regard, a study from Mexico conducted a post-BAE assessment at 24 h and 30, 120, and 180 days [23]. They reported that bleeding was the cause of death in 3 out of 24 patients within 24 h, and surgical resection was required in 4 of the 16 patients who did not have recurrent hemoptysis at the end of 180 days. Survival rates of 24 patients at 30 and 180 days were 75% and 67%, respectively [23]. In contrast, a study from India of 280 patients with a history of tuberculosis reported that immediate control of bleeding by BAE was observed in 255 patients (91%), recurrence of hemoptysis in 18 patients (6%) within 3 months, and in 10 patients within 6 months (3.5%) [24]. The current study’s, immediate control rate of bleeding was 84.8%. Long-term follow-up of the 64 patients showed that 35 patients (54.7%) had no recurrence of hemoptysis for up to 36 months after BAE. However, 29 patients (45.3%) had recurrent hemoptysis after BAE treatment, with a median length of 10 months (2 days to 36 months), and 93.1% of the recurrent hemoptysis occurred within 2 years after BAE. Furthermore, we found that comorbidities, pituitary hormone treatment, the angiographic appearance of arterial dilation and hypertrophy, and materials used for the BAE were significantly associated with the success rate of BAE. At the same time, the etiology of hemoptysis, smoking, and amount of bleeding was not significantly related with the success rate of BAE.

A lesson from our study, and other studies, was that BAE did not guarantee complete success in all patients, with immediate control of bleeding after BAE not being achieved in all patients. Furthermore, some patients had recurrent bleeding despite adequate embolization of the arteries. In this context, it has been reported that the immediate control rate of hemorrhage ranged from 77–100% [5, 6, 10, 26–28], and recurrent rate of bleeding after BAE treatment was 9–44% [5, 6, 10, 11, 20, 22, 26, 28, 29]. Two peak times of recurrent hemoptysis were observed: the first was–1–2 months after BAE, and the second 12–24 months after the procedure [11]. The cause of recurrent bleeding after BAE could be due to the following reasons: incomplete embolization of the bronchial arteries, the presence of non-bronchial systemic arteries (aberrant origin of the bronchial artery), recanalization of embolized arteries, or collateralization caused by continued inflammation [22]. Here, we report that comorbidities, pituitary hormone treatment, and hypertrophied bronchial artery may be related to BAE failure within 24 h. Moreover, pituitary hormone treatment and arterio-arterial or arteriovenous fistula may be risk factors that contribute to the recurrence of hemoptysis after BAE treatment. While the association between pituitary hormone treatment and the risk of hemoptysis recurrence remains to be further investigated, an imperfect BAE procedure due to vasoconstriction of the bronchial artery by pituitary hormone may be the cause of hemoptysis recurrence.

Coils, polyvinyl chloride particles, Gelfoam, and gelatin sponge particles are commonly used in BAE. With newer materials available for embolization and increasing experience with BAE, the complication rate of BAE has gradually diminished over the years, and the rate of major complications remains negligible, with a median incidence of 0.1% (0%-6.6%) [6]. In this regard, the following complications have been reported in the previous studies: chest pain, fever, nausea, and arterial dissection of the patients [26]. Consistently, the complication rate after BAE in the current study was low and did not affect the overall outcome of the therapy.

Although the current study’s findings indicate that BAE is effective for management of massive hemoptysis, this study has some limitations. First, only patients with massive hemoptysis were included in this study. Thus, the therapeutic effect of BAE in patients with moderate or mild hemoptysis, including those with lung cancer, remains to be evaluated. Second, the sample size of the study was small.

In summary, BAE is an effective therapeutic procedure to control acute and massive hemoptysis, with a technical success rate of 81–100% in a recent systemic review [6]. However, recurrence of hemoptysis after BAE may occur for various reasons. The current study’s findings indicate that it is important to treat underlying pulmonary diseases and comorbidities to increase the success rate of BAE and decrease the risk of recurrent bleeding after BAE.

## Data Availability

The datasets used and/or analyzed during the current study are available from the corresponding author upon reasonable request.

## Abbreviations

BAE: Bronchial artery embolization
HRCT: High-resolution CT
CTA: CT angiography
IQR: Interquartile range
OR: Odds ratio
HR: Hazard ratio
